# Physiological and Molecular Aspects of Tolerance to Environmental Constraints in Grain and Forage Legumes

**DOI:** 10.3390/ijms160818976

**Published:** 2015-08-13

**Authors:** Adnane Bargaz, Mainassara Zaman-Allah, Mohamed Farissi, Mohamed Lazali, Jean-Jacques Drevon, Rim T. Maougal, Georg Carlsson

**Affiliations:** 1 Department of Biosystems and Technology, Swedish University of Agricultural Sciences, Box 103, SE-23053 Alnarp, Sweden; E-Mail: georg.carlsson@slu.se; 2 International Maize and Wheat Improvement Center (CIMMYT), Southern Africa Regional Office, MP163 Harare, Zimbabwe; E-Mail: z.mainassaraabdou@cgiar.org; 3 Polyvalent Laboratory for Research & Development, Polydisciplinary Faculty, Sultan Moulay Sliman University, 23000 Beni-Mellal, Morocco; E-Mail: farissimohamed@gmail.com; 4 Faculté des Sciences de la Nature et de la Vie & des Sciences de la Terre, Université de Khemis Miliana, 44225 Ain Defla, Algeria; E-Mail: m.lazali@yahoo.fr; 5 Unité mixte de recherche, Écologie Fonctionnelle & Biogéochimie des Sols et Agroécosystèmes, Institut National de la Recherche Agronomique, 34060 Montpellier, France; E-Mail: drevonjj@supagro.inra.fr; 6 Laboratoire de génétique Biochimie et biotechnologies végétales Faculté des Sciences de la Nature et de la Vie, Université des frères Mentouri, 25017 Constantine, Algeria; E-Mail: maougalr@gmail.com

**Keywords:** abiotic constraints, drought, legume, phosphorus, salinity

## Abstract

Despite the agronomical and environmental advantages of the cultivation of legumes, their production is limited by various environmental constraints such as water or nutrient limitation, frost or heat stress and soil salinity, which may be the result of pedoclimatic conditions, intensive use of agricultural lands, decline in soil fertility and environmental degradation. The development of more sustainable agroecosystems that are resilient to environmental constraints will therefore require better understanding of the key mechanisms underlying plant tolerance to abiotic constraints. This review provides highlights of legume tolerance to abiotic constraints with a focus on soil nutrient deficiencies, drought, and salinity. More specifically, recent advances in the physiological and molecular levels of the adaptation of grain and forage legumes to abiotic constraints are discussed. Such adaptation involves complex multigene controlled-traits which also involve multiple sub-traits that are likely regulated under the control of a number of candidate genes. This multi-genetic control of tolerance traits might also be multifunctional, with extended action in response to a number of abiotic constraints. Thus, concrete efforts are required to breed for multifunctional candidate genes in order to boost plant stability under various abiotic constraints.

## 1. Introduction

The many ecosystem services (biologically fixed nitrogen, soil fertility improvement, health-promoting sources of protein, N-rich green-manure; diversified agriculture *etc.*) that grain and forage legumes provide are often compromised by their sensitivity to stressful conditions causing low yield stability. Moreover, at least 50% of the production of major crops, including legume, is estimated to be lost due to increased frequency of abiotic constraints such as heat, cold, drought, salinity, and low soil fertility [1]. In addition to increased soil salinity, low nutrient (notably phosphorus, P) and water availability are among the most important abiotic constraints affecting legume productivity, especially in arid and semi-arid regions [2,3,4,5,6].

Under these stressful conditions, the occurrence and interactions of molecular and physiological changes at different levels (transcriptomic, metabolomic, cellular, and biochemical) make plant responses highly complex, and even more so as when plants experience both abiotic and biotic constraints. Consequently, plant responses to constraints often involve intricate networks of adaptive, defensive or protective responses. To shed light on legumes’ adaptive responses to abiotic constraints, this review covers biochemical and molecular processes involved in legumes tolerance associated to P limitation, drought and salinity. A better understanding of these processes will be valuable input for strategies to improve the symbiotic nitrogen fixation (SNF) and enhance sustainable cropping systems.

Given their importance for promoting sustainable agriculture, legumes’ sensitivity and adaptive responses to environmental constraints need to be more deeply explored. This will help in developing more effective strategies to improve stress tolerance and subsequently sustainable crop production in increasingly degraded ecosystems. This review aims to highlight the biochemical and molecular mechanisms involved in legumes tolerance to abiotic constraints. Recent knowledge on grain and forage legumes will be explored focusing on the below-ground (roots, nodules, rhizosphere) soil–root interface in order to understand the mechanisms involved in mitigating factors such as drought, salinity and nutrient deficiency (with emphasis on P deficiency).

## 2. Legumes with Nutrient Deficiencies: Case of P-Deficiency and Examples of Adaptive Strategies

Legumes have higher P requirements than non-legumes, especially *in situ* ations where legume nitrogen nutrition depends on the symbiosis with N_2_-fixing rhizobia [7,8,9]. This high P requirement increases the sensitivity of legumes to P-deficiency, a major limiting factor for legume production particularly in acidic and calcareous soils. Under P-deficiency, numerous transcriptional, biochemical, physiological, and morphological responses are triggered to stimulate either the root’s extracellular abilities to acquire rhizosphere soil P or to optimize its intracellular use efficiency and allocation through all plant organs [10,11,12]. Enhanced activity of acid phosphatases (APase) to acquire and remobilize inorganic P (Pi) from organic compounds is one of the most important strategies for improving overall crop P nutrition. The release of APase to the rhizosphere is a typical P-starvation response in higher plants, including N_2_-fixing legumes such as *Glycine max* (soybean) and *Lupinus albus* (white lupin) [13,14,15]. In these legumes, APase activity increased steadily during both root and nodule development and reached a peak in the mature stage, suggesting that this enzyme is a key component for functional nodules [16,17]. The solubility of P has also been reported to be enhanced in both alkaline and acid soils due to the release of protons associated with the exudation of organic acids such as citrate, malate, and oxalate [18,19,20].

### 2.1. Acid Phosphatases: Expression and Activity

In many legumes, the root hairs are not only supplying water, nutrients and exudates, but also play an important role as the primary site for rhizobial infection, leading to the formation of N_2_ fixing nodules. Studies on *G. max*, *Medicago truncatula* (medic) and *Lotus japonicus* (trefoil) reported that nodules, much like flowers, pods and seeds, contain high levels of tissue-specific genes [21,22,23]. In *G. max*, a large number of tissue-specific purple acid phosphatase (*Gm*PAP) genes have been found in both nodule and flower tissues, and these genes were highly inducible under P deficiency [24]. Li *et al.* [24] provided the first evidence that, in addition to their role in P acquisition/recycling in plant tissues, members of the *Gm*PAP gene family are involved in symbiotic interactions with rhizobia or arbuscular mycorrhizal fungi under low P availability. For instance, the PAPs “*Gm*PAP16” and “*Gm*PAP30” had the highest tissue-specific expression in nodules in comparison with other plant tissues [22,23]. Moreover, a putative APase (Nodulin*Pv*NOD33) in mature nodules of *Phaseolus vulgaris* (common bean) was induced during nodule development and is possibly involved in carbon metabolism [25]. Also, it has been found that increased acid phosphatase activities in nodules, roots, and rhizosphere soil of *P. vulgaris* was positively correlated with enhancement of P utilization and homeostasis as a strategy to tolerate P-deficiency [26,27,28,29].

**Figure 1:**
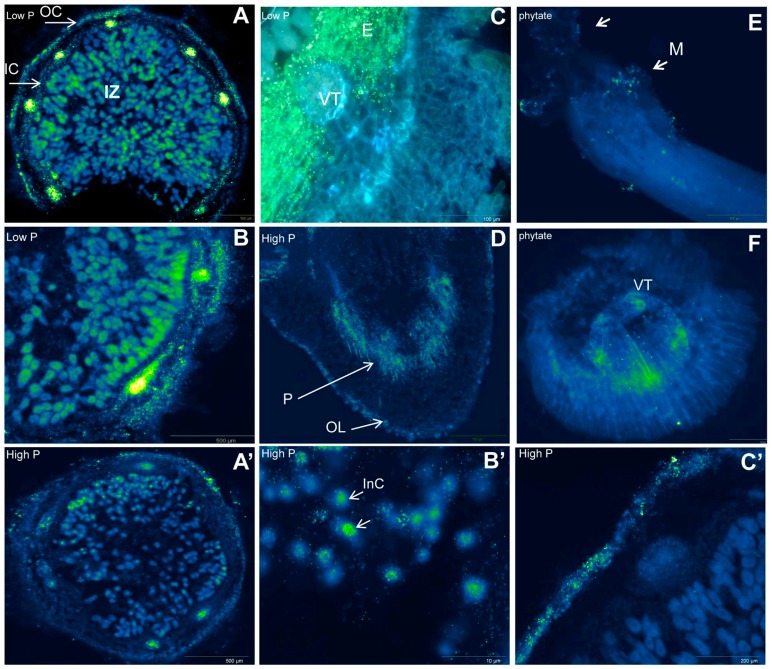
*In situ RT-PCR* of acid phosphatases transcripts (localization and distribution) in cross sections of nodules and roots of *P. vulgaris* grown under P-deficiency (low P, 75 µM·P·plant^−1^). (**A**) Phosphoenolpyruvate phosphatase (500 μm); (**B**) trehalose 6-P phosphatase (500 μm); (**C**) phytase transcript (100 μm); (**D**) phytase transcript in radicle (500 μm) of germinated seeds (**A**–**D** under P-deficiency); (**E**) beta propeller phytase (BPP) transcripts in root tip mucilage (500 μm); and (**F**) cross section of root tips (200 μm) of *P. vulgaris* inoculated with *Bacillus subtilis* under phytate supply. Controls under P-sufficiency (high P, 250 µM·P·plant^−1^) correspond to (**A’**) (500 μm) and (**B’**) (10 μm) for phosphoenolpyruvate phosphatase and (**C’**) 200 μm for nodule phytase. Abbreviations: In nodules: E, Endedormis; IC, inner cortex; InC, infected cells; OC, outer cortex; IZ, infected zone; VT, vascular trace parenchyma; In seeds: OL, outer layers; M, mucilage; P, parenchyma. Images (**A’**–**C’**,**A**,**B**); (**C**,**D**); and (**E**,**F**) provided by Bargaz A, Lazali M, and Maougal T.R, respectively.

Among the diversity of phosphatases, phytase is the only enzyme that has the specific capacity to degrade phytate (C_6_H_18_O_24_P_6_), yielding a series of lower phosphate esters of myo-inositol and Pi [30,31]. Phytate, or phytic acid, is a major constituent and stable form of soil organic P (comprises up to 60%–80% of the soil total P) and seeds (major storage form of P that may account up to 65%–85% of seed total P) [30,32,33]. Seed P remobilization by phytase has been shown to positively influence the establishment and development of the rhizobial symbiosis [34,35]. Utilizing reverse transcription polymerase chain reaction (RT-PCR), Lazali *et al.* [29] characterized the *in situ* localization of phytase transcript, which showed high expression in *P. vulgaris* seeds (Figure 1D). This study demonstrated that the most intensive phytase gene expression was located in the embryo and cotyledons, while lower expression was found in radicles. This seedling phytase gene has also been reported to exhibit high homology (90%) with *Gm*PAP02 while nodule phytase cDNA displayed 94% homology with *Gm*Phy07 [29]. However, phytase enzyme activity seemed to be tissue-specific and likely to vary from seeds to nodules as seed phytase activity was almost 10 times higher than in nodules [29]. Similarly, Li *et al.* [24] found the phosphatase genes, “*Gm*PAP14”, to be specifically expressed in the seeds while Libault *et al.* [22] and Severin *et al.* [23] have found them to be highly expressed in roots. The sub-cellular localization of APase transcripts, especially phytase, has led to co-localization with mineral nutrients (Mg, Ca, K, Fe, Zn and Co) in both *P. vulgaris* seeds [36,37,38] and nodules (for Pi, Fe and K) of the N_2_ fixing legume *Virgilia divaricate* [39]. Based on these findings, it is likely that the low concentration of Pi (co-localized with Fe) in the infected zone of *V. divaricate* nodules [39] under P limitation would be linked to the higher nodule APase activity.

The clustered-root (or proteoid roots) legumes *L. albus* and *L. angustifolius* were also described to efficiently use soil phytate by promoting the rhizosphere microorganisms such as *Burkholderia* species that express high phytate utilization ability [20,40]. In rhizosphere soil, bacteria (except rhizobia) belonging to *Pseudomonas* sp. [41], *Enterobacter* sp. [42], and *Bacillus* sp. [43] have all been reported to exhibit phytase activities with high potential to catabolize phytate as a P source. Localization of APase candidate genes using the *in situ* RT-PCR technique has allowed visualization of the expression of β propeller phytase gene (BPP; bacterial phytase from the genus *Bacillus*) on the mucilage of *P. vulagris* root tips (Figure 1E) inoculated with *B. subtilis* and supplied with phytate [44]. A BPP transcript was also detected in cells inside the roots (Figure 1F), which suggests that *B. subtilis* may either exude its phytase enzyme in the *P. vulgaris* endo-rhizosphere or act as an endophytic BPP-harboring bacterium [44].

Furthermore, recent analyses of several APases have shown that the localization of tissue-specific cDNA of phosphoenolpyruvate phosphatase (Figure 1A) and trehalose 6-P phosphatase (Figure 1B) were mainly expressed in infected cells and nodule cortex of *P. vulgaris* [27,28]. These studies provided the first evidence that phosphoenolpyruvate phosphatase and trehalose 6-P phosphatase were differentially expressed among nodule tissues of two recombinant inbred lines of *P. vulgaris* (RIL 115 and RIL147), and suggested the abundance of their transcripts in infected cells at the vicinity of inner- and in outer-cortex cells to be involved in the adaptation to P-deficiency. This overexpression under P-deficiency was coupled with increased enzyme activity, improved symbiotic efficiency and increased N_2_ fixation and seems to play an important role in tolerance to low P availability [26,27]. Coincident with these findings, abundance of phytase cDNA was significantly overexpressed in *P. vulgaris* nodules (Figure 1C) of the P-efficient RIL115 compared to the P-inefficient RIL147 [45]. In nodules of both RILs, the abundance of phytase transcripts was higher in the outer cortex than in infected cells [45]. This result highlights an important intra-nodular phytase distribution and suggests a functional role in supplying adequate amount of Pi for nodule functioning [45]. These results are in agreement with Li *et al.* [24] on the overexpression of a large number of *Gm*PAP genes in P-deficient nodules of *G. max*. These *Gm*PAP genes were markedly increased (66%) under P-deficiency as compared to the *Oryza sativa* (rice) *Os*PAP (48%) and *Arabidopsis thaliana At*PAP (14%) genes [46,47,48]. Moreover, several studies have revealed that APases existed in the symbiosome membrane of *G. max* and were possibly involved in P homeostasis within the nodules [16,49].

### 2.2. Organic Acid Exudation

With a high affinity for divalent and trivalent cations, negatively charged organic acids displace P from insoluble complexes and improve its availability for plant uptake in acid or alkaline soils [50]. Malate exudation is simultaneously coupled with proton release, and the stimulation or repression of the gene encoding an ATPase involved in proton release (plasma-membrane-bound ATPase) also regulates citrate release, indicating a link between citrate exudation and proton efflux [51]. Exudation of organic acids in the rhizosphere is likely to be accompanied by improved availability and uptake of P as well as di- and tri-valent cations [18,52]. This release of organic acids in the rhizosphere is known to be substantial in several lupine species. For example, the cumulative citrate exudation from the cluster roots of *L. albus* was equivalent to 23% of the total plant dry weight [18,53]. Besides the documented benefit of carboxylate exudation under P-deficiency, the magnitude of this exudation in white lupine has also been reported to be highly important with iron deficiency [54]. Likewise, it was postulated that the P-deficiency-induced carboxylates in white lupine might increase the availability of other micronutrients such as copper and zinc, although their deficiency (especially zinc) does not stimulate cluster-root formation or carboxylate release [55,56].

In legumes, specific forms of malate dehydrogenase (MDH) and phosphoenolpyruvate carboxylase (PEPC) genes have been reported to be highly expressed in root-nodules (5- to 15-fold as compared to other tissues), and have an important role in supporting bacteroid respiration and nodule N_2_ fixation activity [57,58]. A study by Tesfaye *et al.* [59] has shown that enhanced organic acid exudation resulted in an improved tolerance to Al toxicity and enhanced P uptake of transformed *Medicago sativa* (lucerne, alfalfa) plants with nodule-enhanced forms of malate dehydrogenase (neMDH) cDNA. This study has reported a 1.6-fold increase in root tip MDH enzyme activity along with a significant increase in root exudation of several organic acids (*i.e.*, citrate, oxalate, malate, succinate, and acetate) as compared to non-transformed *M. sativa* [59]. Similarly, recent findings by Liang *et al.* [60] have suggested that *G. max* adaptation to both P-deficiency and Al toxicity is likely due to higher root malate exudation. The expression of *Gm*ALMT, a *G. max* malate transporter gene, in roots of the P-efficient *G. max* “HN89” has been found to be pH dependent, and mainly enhanced by P and Al availability. Several studies have also suggested that malate exudation could potentially be a response to acidic soil conditions, Al toxicity or P limitation [61,62,63]. In non-legumes, other ALMT genes have been characterized to play important roles in guard cell anion channels for organic anion transport and regulation of cytosolic malate homeostasis such as *At*ALMT9, *At*ALMT12 in the vacuole of *A. thaliana* [64] as well as the opening/closure of stomatal complexes in *Hordeum vulgare* (barley) leaves (*Hv*ALMT1) [65,66]. Exuded carboxylates can be catabolized by rhizosphere microorganisms, thereby limiting the benefit of carboxylate-mediated solubilization of P and some micronutrients [67,68]. It has been shown that beneficial rhizosphere microorganisms may stimulate the exudation of carboxylates, as in the case of sorghum root malate exudation that was induced by efficient colonization of N_2_ fixing free-living bacteria [67,69]. As a potential strategy to avoid microbial breakdown of released organic acids, *L. albus* can exude flavonoids, which have an antimicrobial activity against fungal pathogens [68,70]. For instance, isoflavone prenyltransferase activity resulting from expression of the isoflavonoid prenyltransferase gene “LaPT1” in *L. albus* roots has been shown to be antimicrobial [71] and play a key role in limiting the microbial breakdown of citrate [68,72]. Root-secreted malic acid was also reported in non-legume plants such as *A. thaliana* and described to promote beneficial rhizosphere soil bacteria such as *B. subtilis*, which showed higher root colonization in response to shoot infection by pathogenic bacteria [73].

### 2.3. Phosphorus Use Efficiency Involves Complex Quantitative Traits

Tolerance to P-deficiency, via enhanced P acquisition or internal use efficiency, is expressed as continuous (quantitative) phenotypes, *i.e.*, under control of multiple genes. Adaptive phenotypes or responses such as stimulation of phosphatase activity and organic acid exudation or modified root architecture (root length, root hair density, branching and length) are thus expressed in numerous traits. Genetic control of the traits may be revealed through molecular markers approaches such as quantitative trait loci (QTLs) analysis [74,75]. Several studies have shown the importance of root architecture (basal and adventitious roots, length and root hair density), organic acids and H^+^ exudation for P acquisition using QTL analysis [74,75,76,77]. In this context, QTLs have been identified for P acquisition or P use efficiency in, e.g., *G. max* [78], *O. sativa* [79], *P. vulgaris* [76,80], *Triticum aestivum* (wheat) [81] and *Zea mays* (maize, corn) [82]. However, it seems difficult to identify the candidate genes that are implicated in P use efficiency in most identified QTLs, as P acquisition/ use efficiency is most likely under the control of multiple genes [83].

Searching and selection for root trait QTL markers associated with higher shallow rooting at the nutrient-rich topsoil layers might enhance P acquisition, given that P availability is greater in upper as compared to deeper soil layers. Shallow-rooted genotypes of *P. vulgaris* with more gravitropic plasticity were indeed associated with higher plant growth and P uptake under low P conditions in the field [77]. A study by Liao *et al.* [77] mapped 16 QTLs controlling numerous shallow-root traits that were associated with higher P acquisition efficiency under low P conditions. A further study indicated that basal root growth has a central role to improve P acquisition in *P. vulgaris*, based on the link found between more than 20 QTLs of basal root growth/development and the QTLs for P uptake efficiency in field-grown *P. vulgaris* [74]. In comparison to non-legume plants, Reymond *et al.* [84] found three QTLs that explained most of the variation associated with primary root length response to low P availability in a RIL population of *A. thaliana*. Advanced studies by Zhang *et al.* [85] have shown an acid phosphatase-encoding gene, “*Gm*ACP1”, to be located within the major QTL qPE8 (a highly significant region on chromosome 8) which is involved in P use efficiency in *G. max*. Likewise, the expression quantitative trait loci (eQTL) mapping for Pi transporter *Gm*PT1 (on the chromosome 10 “*Gm*10 (LG-O)) was associated with seed yield, PUE and photosynthetic rate in *G. max* [86]. This study also described that *GmPT1* was markedly expressed under long-term P starvation in older leaves and highly induced in meristematic tissues of leaves and lateral roots. The specific expression of *GmPT1* suggests a key role in the remobilization of Pi within *G. max* plants, and a similar role has previously been reported in *H. vulgaris* and *A. thaliana* whose low-affinity Pi transporters, *HvPht1;6* and *AtPht1;5*, were overexpressed under P-deficiency [87,88]. Furthermore, a study by Yan *et al.* [76] identified QTLs associated with root hair and organic acid exudation in a RIL population derived from the cross of two contrasting *P. vulgaris* genotypes, DOR364 and G19833. In this study, 19 QTLs associated with root hair, acid exudation and P-uptake traits were detected and at least three loci (*Tae4.1*, *Hex4.1* and *Hex10.1*) were associated with P uptake. The continued advancement of knowledge about QTLs associated with efficient P acquisition and/or use in N_2_-fixing legumes will improve our understanding of the genetic control of these traits, and opens up possibilities for P-efficient legume genotypes via e.g., marker-assisted breeding and selection.

## 3. N_2_-Fixing Legume and Micronutrient Deficiencies

In addition to the essential mineral nutrients required for normal growth and development of plants, mineral nutrition is critical in legumes for the successful establishment and functioning of symbiotic root nodules. Iron (Fe), Boron (B) and Molybdenum (Mo) seem to be the most important micronutrients in N_2_ fixing legumes. For instance, Fe is required for the synthesis of iron-containing proteins in the host plant and a key constituent of leghemoglobin and nitrogenase, therefore playing a vital role in the SNF [89,90,91]. The rate of N_2_ fixation in *P. vulgaris* nodules was reported to be positively correlated with increasing nodule Fe concentrations [92]. In addition, Fe is also required for the synthesis of cytochromes, ferredoxin, and hydrogenase [93,94,95,96]. Boron was also found to be crucial to several stages of legume root nodule development such as the establishment of the symbiosis in term of nodule structure and function [97,98], bacterial–plant molecular signaling, rhizobial infection, and maturation of the N_2_-fixing symbiosomes [98,99]. In legumes, Mo acts as a cofactor for the nitrogenase enzymes [100] and is crucial for the synthesis of proteins associated with N metabolism [101]. Several soil factors influence solubility and availability of micronutrients, notably pH, cation exchange capacity, organic matter, CaCO_3_ content, soil texture and moisture. Under adverse soil conditions, legume plants need to develop different strategies in order to withstand the stress and optimize their use of limited essential nutrients.

### 3.1. Tolerance Mechanisms Associated with Fe Deficiency

In soils, Fe exists mainly in chemical associations with hydrogen oxide, phosphate, and various deposited compounds [102]. Total Fe concentrations in soil might vary from 1%–20%, but soil Fe is often present in insoluble forms, and Fe availability often does not correspond to the optimal concentration for plants even though optimal plant intracellular Fe concentration is only around 0.005% [103]. In nodulated legumes, Fe deficiency is very common in alkaline soils, and its negative effects have been reported in *Cicer arietinum* (chickpea) [104], *P. vulgaris* [105], *Arachis hypogea* (peanut) [94], and *Lupinus* spp. [106]. The legume–rhizobia symbiosis is particularly sensitive to Fe deficiency as it may not only limit host-plant growth but also root nodule bacterial survival and growth, nodule formation and function [107,108]. To cope with Fe-deficiency, two main strategies have been developed by plants to mobilize and acquire Fe from soil; (i) the “Strategy I” involving morphological and physiological responses by plant roots; and (ii) the “Strategy II” involving the exudation of phytosiderophores.

“Strategy I” implies numerous morphological and physiological changes initiated under Fe-deficient conditions and include sub-apical swelling with the proliferation of root hairs, development of transfer cells, induction of ferric chelate reductase activity, acidification of the rhizosphere, organic acid release [109], and up-regulation of Fe(II) transporters [110]. The transfer cells have been shown to occur in roots of many Fe-efficient plant species, and are most likely the sites of Fe deficiency-induced root responses of the Strategy I [111,112,113,114]. It has also been shown that transfer cells may be induced either in the rhizodermis or in the hypo-dermis, and are characterized by wall ingrowths in order to increase the membrane surface area, as well as the stimulation of ion uptake sites, H^+^-ATPases, sucrose and amino acid transporters [114,115,116,117].

Enhancement of Fe uptake was associated with induction of both H^+^ secretion and the activity of Fe(III) reductase in the rhizosphere of Fe-deficient *G. max* [118]. A study by Slatni *et al.* [119] reveals that the induction of root Fe(III) reductase activity is necessary for Fe uptake and can be coupled to the rhizosphere acidification capacity linked to the H^+^-ATPase activity. Morphologically, an increase in root hair length under low Fe concentrations increases Fe uptake efficiency and is considered to be a growth strategy by many plant species in order to survive under Fe-limited conditions. This increase in root hair length and number of transfer cells is found to be positively correlated with the amount of detectable H^+^-ATPases [120].

“Strategy II” is more specific to Fe deficiency with the involvement of highly specific uptake systems for Fe(III)–phytosiderophores [121]. Diverse types of siderophores are produced by different rhizobial species. *Rhizobium leguminosarum* is known to synthesize a cyclic trihydroxamate type siderophore (vicibactin) and *Sinorhizobium meliloti* produces rhizobactin 1021, a dihydroxamatesiderophore, under Fe deficiency [122]. Catecholatesiderophores are synthesized by rhizobia from the *Vigna unguiculata* group [123] while salicylic and dihydroxybenzoic acids are produced by *Rhizobium ciceri* isolated from *C. arietinum* nodules [124]. In addition, Fe-deficiency adaptations belonging to these two strategies were reported to be mainly regulated by ethylene and/or auxin signaling [120], with ethylene mainly associated with Fe-deficiency response in strategy I plants. Similarly, a number of phytohormones are found to likely be involved in the regulation of Fe deficiency responses, including ethylene [125,126], cytokinins [127], and brassinosteroids [128].

In order to protect its symbiotic organs against low Fe availability, the N_2_-fixing legumes likely need to develop mechanisms involving both the acquisition and utilization of Fe. Concerning the utilization of Fe, several studies suggest that enhanced allocation of Fe to nodules and the improved Fe use efficiency for nodule growth and N_2_ fixation were the basis for the tolerance of *P. vulgaris* cultivars to Fe deficiency [92,129].

### 3.2. Boron and Molybdenum

Boron availability in soil decreases under alkaline conditions (especially with calcium carbonate that acts as an important B adsorbing surface), where intracellular B concentrations might fall below 10 ppm in young leaves, indicating that the plant suffers from B deficiency [97,98]. Early rhizobia-root interactions, infection, cell invasion [98], symbiosome development [99,130], and nodule organogenesis [131] are all greatly affected by B deficiency. B deficiency leads to a high reduction in nodule number and nitrogenase activity [132], caused by the repression of nod gene activity of root exudates from B-deficient legumes [98]. Since this micronutrient is not required for rhizobial growth, the strong effect of B on legume–rhizobia symbioses has always been related to the structure and stability of plant-derived components crucial for nodule development [133]. Under conditions of B-deficiency, enhanced translocation of B from the root system to leaves usually takes place, and genotypic differences in B re-translocation usually reflects a tolerance mechanism to B deprivation. Searching for mechanisms related to B-deficiency tolerance in non-legume plants, Kobayashi *et al.* [134] identified 13 genes whose expressions were up-regulated in low-B-acclimated *N. tabacum BY-2* cells, using cDNA differential subtraction. The induction of these genes that mainly encode the oxidative stress-responsive enzymes (such as glutamine synthetase, class tau glutathione *S*-transferase, and glucosyltransferase) point out the effect of B-deficiency to induce cell oxidative damage that was presumably caused by impaired cell wall structure [134].

Molybdenum is a trace element found in soil at very low concentrations, usually around 1–36 ppm [114,135], and even if the critical Mo concentration for plants is usually below 0.2 ppm, deficiency of this micronutrient is a widespread agricultural problem, especially in acid soils [114]. In legumes, Mo is required for the synthesis of proteins associated with nitrogen metabolism [100], and Mo acts as a cofactor for the nitrogenase enzymes [101]. In general, it has been shown that Mo availability for plant growth decreases with decreasing soil pH (nine-fold lower in *G. max* shoots from pH 7 to pH 5), a soil factor that leads to decreased plant Mo concentration and thus reduced N_2_-fixing activity [114,136,137]. Maintaining an adequate Mo concentration in nodules under Mo-limiting conditions may be a strategy for optimal nitrogenase activity, but may also be valuable for plant growth and yield if the aboveground allocation of Mo was not affected. Furthermore, changes in root morphology, including an increase in the absorption area of the total root surface, root length and number, can mediate the adaptation of plants to soil Mo-deficiency. According to Nie *et al.* [138], a genotype may be Mo efficient or inefficient due to one or more of the following mechanisms: higher Mo root uptake abilities from soil, the manner in which Mo is transferred, and its assimilation within the plant. For a given species, the mechanism responsible for Mo efficiency may differ between genotypes and with the extent of deficiency.

## 4. Drought Tolerance in N_2_ Fixing Legumes

### 4.1. Overview on the Impact of Drought on Legumes

Drought affects both legumes and the symbiotic rhizobia, which ultimately limit the SNF. However, this effect may depend on the relative influence of each symbiotic partner on the regulation of SNF. Initiation, development and activity of nodules were reported to be more sensitive to drought than general root and shoot metabolism. In addition, drought constraint imposed during vegetative growth seems to be more detrimental to nodulation and nitrogen fixation than if imposed during the reproduction stage [139]. Infection and nodulation can be reduced or even suppressed by water deficit due to modifications of rhizobial cells and decreased number of infection threads formed inside root hairs [140]. It has also been shown that, during flowering or the grain-filling period, water shortage has a dramatically negative impact on the final legume seed yield [141,142]. Water uptake/use and its temporal pattern are crucial for crops grown with a limited amount of water in the soil profile because crop reproductive success depends largely on a sustained water use into the reproductive growth stage [143,144].

Plants use various mechanisms to cope with drought constraint. Extensive root development may lead to drought avoidance by enhanced extraction of soil water, and was related to maintaining seed yield despite drought during the terminal growth stages in several grain legumes [145,146]. At the root level, proteome analysis at different stages of drought stress revealed that proteins associated with cell signaling (lectins and oxidoreductases) were significantly up-regulated during drought stress [147]. Proteins that degrade or detoxify reactive oxygen species (ROS) play important roles in protecting essential plant functions against drought-induced oxidative damage [148], maintaining intracellular redox homeostasis or mediating redox signaling for induction of specific stress responses [149]. Besides, key enzymes of sulphur metabolism and proteins associated with root structure (tubulin) were reported to be down-regulated by drought stress while the expression of enzymes associated with root morphology (actin) was up-regulated [147]. The alteration in the expression of these proteins was assumed to have a positive correlation with the root architectural modifications, which in turn may have an indirect effect on the overall plant photosynthetic process, due to the alterations in the net water conductance [150].

Plant–water homeostasis is also regulated by a group of proteins called aquaporins. These proteins, which include nodulin-26-like intrinsic proteins initially identified in symbiosomes of legumes, are specialized in water transport and therefore may play a critical role in plant adaptation to water deficit through water uptake [151]. Aquaporins also function to modulate abiotic constraints-induced signaling. Their versatile functions have made aquaporins suitable candidates for development of transgenic plants with increased tolerance to different abiotic constraints, including drought [152].

### 4.2. Overview of the Regulation of SNF under Drought

The regulation of SNF under drought was reported to be governed by various factors including internal oxygen availability, carbon flux within nodules and N-feedback regulation [18,153,154,155,156,157]. Despite recent progress in the field, the molecular mechanisms responsible for these physiological responses and their interactions are not yet fully understood.

A number of studies have shown that SNF can be locally regulated under drought stress in soybean [157,158], *Pisum sativum* [159] and *M. truncatula* [153]. Using a split-root system that allows differentiation between local and systemic responses, Gil-Quintana *et al.* [153] analyzed the variations in the content of amino acids and ureides in different plant organs, and measured the levels of ureide metabolism enzymatic activities in nodules of drought-stressed *G. max*. Their results support the hypothesis of a local regulation of SNF. In addition, ureide accumulation may be a more widespread response to water deficit not necessarily related to the regulation of SNF, since nitrate-fed *G. max* plants showed some level of accumulation when exposed to drought [160]. Recent studies have shown that the ureide accumulation depend more on the plant developmental stage than on the growth conditions [161].On the other hand, drought-induced declines of nitrogenase activity has been reported to be caused by N-feedback inhibition of SNF [156,157], which was related to the accumulation of ureides in leaves [162,163] and nodules [157,158] of *G. max* under different water-deficit treatments. In addition to ureides, other N compounds like asparagine and aspartic acid were also proposed to trigger the inhibition of SNF [157,164]. The drought-induced accumulation of N compounds was associated with reduced transpiration rates due to lower xylem translocation rates as a consequence of the decreased transpiration [165]. However, recent report showed that the accumulation of ureides and reduction of transpiration rates are not correlated under drought constraint [153]. Another origin of this accumulation reside in the decrease of the shoot N demand which has also been shown when inorganic N is applied to N_2_-fixing legumes [166]. Therefore, the ureides’ accumulation does not seem to occur specifically under drought stress. The third possible origin of ureides’ accumulation could be through alterations in the metabolism of ureides [167]. However, ureide catabolism was reported by Gil-Quintana *et al.* [153] to be more affected than the *de novo* synthesis under stressful conditions. These authors have suggested that the observed accumulation of ureides in nodules may be more related to a decline in the activity of degradation rather than to increased biosynthesis.

From a proteome point of view, proteins related to plant metabolism showed a general trend of down-regulation while bacteroid cells were up-regulating protein biosynthesis, probably as an adaptation to the water deficit imposed [168]. Significant changes were reported for the functional group glycolysis/TCA cycle which includes enzymes such as sucrose synthase, fructose-bisphosphate aldolase, phosphoenolpyruvate carboxylase, and malate dehydrogenase [153]. Several metabolic pathways, such as sucrose synthase and glutamine synthetase [154,168], were reported to coincide with the decline in SNF rates. Chalcone isomerase (CHI), a key enzyme in flavonoid biosynthesis known to influence the process of nodulation, was significantly down-regulated by drought, and the expression patterns of CHI were reported to be positively correlated with the concomitant changes in the biomass of root nodules [147].

Regarding the transcriptional regulation of SNF, the reduction of nitrogenase activity under drought constraints was associated with a reduced expression of the nifK gene [169]. According to a recent study by Furlan *et al.* [170], transcript levels of glutathione reductase increased in response to drought with a subsequent increased enzyme activity. Likewise, marker transcripts responsive to drought, abscisic acid and H_2_O_2_ were up-regulated. However, superoxide dismutase and glutathione *S*-transferase activities were unchanged, despite up-regulated gene transcription while increased activity of ascorbate peroxidase (APX) did not seem to be related to changes in cytosolic APX transcript levels [170]. Furthermore, inoculation of *P. vulgaris* plants with a *R. etli* strain having enhanced expression of the cytochrome *cbb_3_* oxidase in bacteroids was reported to reduce the sensitivity of *P. vulgaris–R. etli* symbiosis to drought and can modulate carbon metabolism in nodules [171]. This was related to the high respiratory capacity of the bacteroids given the role of both the high oxygen affinity and the overexpression of *cbb_3_* oxidase in fulfilling high-energy demand for efficient SNF [171,172].

## 5. Salinity Tolerance in N_2_ Fixing Legumes

Salinity negatively affects the legume–rhizobium symbiosis through osmotic and/or ionic effects that inhibit several physiological and biochemical processes and limit host plant growth, nodulation as well as the survival and proliferation of rhizobia [173,174,175]. The prevention from damages caused by salt stress and their repair are necessary for cell survival and plant development. Legumes’ ability to tolerate saline conditions is associated with changes in many physiological and molecular processes, including sequestering of sodium ion (Na^+^), accumulation of osmoprotector solutes, induction of antioxidative stress responses and hormone biosynthesis [174,175,176,177,178]. Understanding legume responses to salinity requires a strong knowledge on all salinity stress-related agro-biological mechanisms as well as determination of existing connections between mechanisms at different levels (morphologic, physiologic, molecular, *etc.*) and different organs such as nodules of the N_2_-fixing legumes. Also, the connection with functional genomics, particularly with the recent advances in genomics and bioinformatics, could lead to the identification of candidate genes as tools to elucidate most mechanisms involved in the efficiency of legume–rhizobium symbiosis under such environmental constraints.

### 5.1. Intracellular Sequestration of Sodium

Plants, as well as legume–rhizobium symbiosis, detect salt stress through the ionic (Na^+^) and osmotic signals. The excess in Na^+^ may be detected by the transmembrane proteins or the Na^+^ receptor proteins [179]. The excess in Na^+^ and Cl^−^ causes changes of protein structures and membrane depolarization which can lead to the perception of the ionic toxicity. In legumes, osmotic adjustment can be achieved by Na^+^ sequestration in nodules, and more so as when free amino acid accumulation and carbohydrate allocation to nodules are also achieved [180]. These authors found that Na^+^ sequestration (besides other mechanisms) in nodules of a salt-treated *M. truncatula* was associated with tolerance to salt stress. Conversely, Krouma *et al.* [181] reported that Na^+^ accumulation in nodules and leaves of *C. arietinum* was associated with sensitivity to salinity, while an increased Na^+^ accumulation in roots may contribute to the osmotic adjustment and tolerance to salt stress. Under saline conditions, vacuolar sequestration of Na^+^ is an important and profitable strategy for osmotic adjustment at the same time that may reduce the cytosolic Na^+^ concentration. The Na^+^/H^+^ antiporters of plasma membrane that pump Na^+^ ions from root cells toward leaves is likely the first line of defense in order to prevent the accumulation of Na^+^ in the cytosol [179]. The vacuolar Na^+^/H^+^ antiporters use the proton gradient generated by the vacuolar H^+^/adenosine triphosphatase (H^+^/ATPase) and H^+^/pyrophosphatase (H^+^/PPase) for Na^+^ sequestration into the vacuole. The activation of the tonoplastic H^+^/ATPase and H^+^/PPase under salt stress, and the coordination between Na^+^/H^+^, H^+^/ATPase and H^+^/PPase antiporters are therefore likely to be crucial for salt stress tolerance [182].

### 5.2. Biosynthesis of Osmoprotectants

Salt-tolerant legumes realize the osmotic adjustment by involving a fine-tuned coordination between physiological mechanisms whose importance appears to vary between roots, nodules and shoots [173,183]. One of the adaptive salinity-tolerance strategies, that maintain the ionic and intracellular osmotic homeostasis, is the accumulation of osmolytes (or osmoprotectant), mainly amino-acids (proline, glycine betaine) and sugars [173].

Several studies have shown a significant accumulation of proline in both shoot and nodules of *M. sativa* that induced tolerance to salt stress [173,184,185]. Differential expression of the proline metabolism genes in *G. max* was investigated under salinity, showing that the expression of the Δ^1^-pyrroline-5-carboxylate synthetase (*GmP5CS*) gene depends on the intensity of salt stress [186]. Over-expression of *P5CS* in a transgenic *M. truncatula* (with *P5CS* gene from *Vigna aconitifolia*) led to proline accumulation in shoots, roots and nodules with positive impacts on osmotolerance, plant growth and nitrogen fixation [187]. Improving nitrogenase activity in this transgenic *M. truncatula* as compared to the correspondent wild type could be attributed to the higher proline accumulation in nodules that would exert a protective effect to both nodule metabolism and N_2_-fixing activity [187]. Similar results were found in *P. vulgaris* that up-regulated the expression of *PvP5CS* in leaves under salinity [188]*.* Overall, proline acts as an osmoticum and its cytoplasmic accumulation neutralizes the ionic and osmotic effects of salt accumulation in the vacuole [187,189]. This amino acid also plays important roles in maintaining the cytosol-vacuole pressure and pH well controlled [190] as well as the stability of membranes [191].

Stimulation of biosynthesis and accumulation of betaines (nitrogenous osmolytes) such as proline (Pro)–betaine and glycine (Gly)–betaine is a valuable strategy to maintain turgor pressure necessary for continued growth under salinity constraint, both in legumes and non-legumes [185,192,193]. Pro–betaine is the main osmoprotectant identified in *M. sativa* [185]. Both increased accumulation and compartmentalization of Pro–betaine and proline highlighted a tolerance trait in a salt-stressed *M. sativa*, especially in nodules where large peri-bacteroid spaces (attributed to an increased turgor pressure) might be due to the elevated Pro–betaine and proline in the cytosol and bacteroids [185]. In *Sinorhizobium meliloti*, the rhizobium species forming symbioses with *Medicago* spp., previous works have demonstrated the important role of Pro– and Gly–betaines for inducing nodulation genes [194] and osmotic stress resistance [195,196]. In the N_2_-fixing *S. meliloti*, Boscari *et al.* [197] identified and characterized the BetS gene which encodes the Gly–betaine/Pro–betaine transporter required under early osmotic adjustment. The *S. meliloti* BetS activity appears to be Na^+^-driven, while its inactivation results in loss of osmoprotection after an osmotic stress [197].

Sugars may contribute to over 50% in the osmotic adjustment of glycophytes subject to salinity conditions [191]. The accumulation of carbohydrates in leguminous plants in response to salinity has been documented in *P. vulgaris* and *P. acutifolius* [198] as well as in *M. sativa* [173]. Their main functions are in the osmo-protection, osmotic adjustment, carbon storage and sequestration of free radicals [191]. The signal generated by signal transduction cascades, starting with sensor proteins that sense the plant cell sugar status, involve mitogen-activated protein kinases, protein phosphatases, Ca^2+^ and calmodulins, resulting in appropriate gene expression [199]. Numbers of genes are either induced or repressed depending upon the status of soluble sugars [200].

### 5.3. Responses of Antioxidant-Gene Enzymes

Like drought stress, salt stress also causes the formation of ROS such as hydrogen peroxide (H_2_O_2_), superoxide (O_2_^−^) and the free radicals. These ROS cause oxidative damage to various cellular components, including membrane lipids, proteins and nucleic acids [201]. To overcome oxidative stress and damages caused by salinity, plants have developed antioxidant non-enzymatic and enzymatic systems [202]. Non-enzymatic processes include binding or scavenging of radicals by β-carotenes, ascorbic acid, α-tocopherol and reduced glutathione. Enzymatic processes are biological reactions involving several enzymes such as superoxide dismutase, guaiacol peroxidase, ascorbate peroxidase, catalase, polyphenol oxidase and glutathione reductase [176,203,204,205]. Superoxide dismutase (SOD) is considered as the first enzymatic system defense against ROS, being responsible for the dismutation of O_2_^−^ to H_2_O_2_ and O_2_. Catalase and peroxidases catalyze the conversion of H_2_O_2_ to water and O_2_ [206].

**Figure 2:**
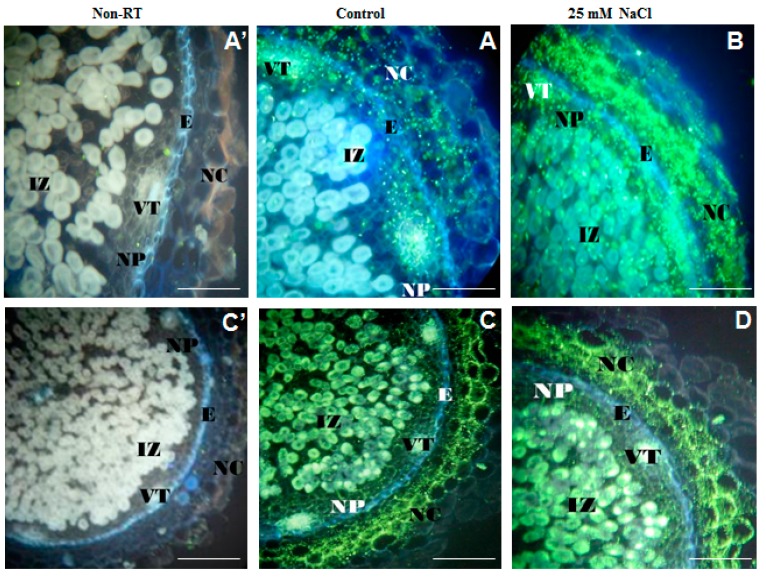
*In situ RT-PCR* localization of ascorbate peroxidase (APX2, (**A**,**B**), 200 μm) and Protein Phosphatase 2C (PP2C, (**C**,**D**), 500 μm) transcripts in cross sections of *C. arietinum* nodules under salinity constraint. Negative controls without reverse transcription (non-RT) are shown in (**A’**) (200 μm) for APX2 and (**C’**) (500 μm) for PP2C. Abbreviations: IZ (infected zone), NP (Nodule parenchyma), E (nodule endodermis) and NC (nodule cortex), VT (vascular trace). Images (**A**,**B**) used from Molina *et al.* [212] under the terms of the Creative Commons Attribution License (http://creativecommons.org/licenses/by/2.0). Images (**A’**,**C’**,**C**,**D**) are provided by Zaman-Allah M.

In *M. sativa*, increased activity of antioxidant enzymes has been considered as an adaptation mechanism to salt stress [203]. Expression of antioxidant genes were also correlated with antioxidant enzymatic activities in salt-treated roots of *M. truncatula* genotypes [207]. Arab and Ehsanpour [208] noted that treatment of *M. sativa* seeds with ascorbic acid increased the level of salt tolerance. Maintaining a high antioxidant activity is positively correlated with decreased lipid peroxidation, stability of nodule’s cell membranes and thereafter maintaining a high nodule biomass. Mahmoudi *et al.* [209] highlighted the higher expression of *Fe-SOD* and *Mn-SOD* genes found in the saline-tolerant *Lactuca sativa* “Verte de Cobham” as an important salt-tolerance trait. Likewise, an increased activity of SOD was detected in salt-stressed nodules of *P. vulgaris*, and reported to be highly involved in tolerance to salinity constraints [210]. Similarly, high peroxidase activity was noted in *C. arietinum*-rhizobium symbiosis that was the most tolerant to salt stress [211]. Nodule cortex/parenchyma of salt-stressed *C. arietinum* exhibited high expression level of antioxidant genes, as for the ascorbate peroxidase “APX2” [212] (Figure 2A,B). These findings suggest a key role for antioxidant genes’ expression, not only for an adequate intra-nodular antioxidant defence, but also for nodule respiration during N_2_ fixation. This study [212] also indicates a powerful scavenging of the potentially harmful hydrogen peroxide (H_2_O_2_), thus preventing oxidative damage in nodules.

### 5.4. Acid Phosphatases under Salinity

Acid phosphatases activity under salinity was shown not to have such a universal response as for low P conditions. Farissi *et al.* [174] reported an increased APase activity in *M. sativa* roots under increasing salinity. However, studies by Faghire *et al.* [178] and Zaman-Allah *et al.* (unpublished data) showed slight changes between salt-treated and control nodules of *P. vulgaris* and *C. arietinum* in terms of intra-nodular APase expression. The expression of a gene coding for the protein phosphatase PP2C-type showed clear differential expression among tissues of *C. arietinum* nodules but did not provide clear differences in response to salinity (Figure 2C,D). However, these slight differences do not exclude that PP2C may react against salinity effects given that members of the phosphoprotein-phosphatases family are known to be involved in the regulation of several signaling pathways, including oxidative stress for phosphotyrosine-phosphatases [213].

On the other hand, an impaired activity of APase such as the inositol-1,4,5-trisphosphate 5-phosphatase was reported in salt-grown roots of *M. sativa* [214]. This low activity was associated with intracellular accumulation of the inositol-1,4,5-trisphosphate (IP_3_) which would act as a soluble secondary messenger molecule implicated in the mobilization of intracellular Ca^2+^ [214]. In addition, the IP3-induced cytoplasmic Ca^2+^ was reported to be involved in signaling pathways and activation of stress responses [215,216,217]. In nodules of various legume species, accumulation of trehalose (a major carbohydrate synthesized from a rapid conversion of trehalose 6-P to trehalose by trehalose 6-P phosphatase) was associated with tolerance to numerous abiotic constraints such as salinity [218,219,220,221,222]. Streeter *et al.* [220,222] suggested that trehalose plays a central role in SNF since trehalose appears in nodules at the onset of N_2_ fixation. In addition, trehalose is thought to be involved in intercellular-spaces occlusion of the nodule cortex with glycoproteins or water [223,224,225]. Trehalose may thus be implied as an osmoticum for the change in morphology of inner-cortex cells, as previously suggested as a mechanism for osmoregulation of the nodule permeability to oxygen [9,226].

### 5.5. Phytohormones in Regulation of Salinity Tolerance

The response of plants to environmental constraints is regulated by phytohormones and plant growth regulators such as abscisic acid (ABA), salicylic acid, and a group of polyamines [227]. For instance, ABA has been shown to play a major role in plant signaling and tolerance to a variety of stresses, including drought, cold, and salinity [228,229,230]. In legumes, several studies have reported that alleviating the damages inflicted under salinity co-occurred with increased ABA contents in various tissues such as nodules of *P. vulgaris* [231], *M. ciliaris* [232] and *M. sativa* [227]. It has also been shown that both salinity and ABA application significantly increased ABA content in salt-stressed *M. sativa* nodules with positive consequences on plant growth and SNF [227]. These authors also found that increasing ABA application under salinity have induced the activity of several nodular antioxidant enzymes such as superoxide dismutase, catalase, and glutathione reductase. Similar results were reported in *P. vulgaris* that has markedly reduced the amounts of malondialdehyde and hydrogen peroxide in response to the combined application of gibberllic and ascorbic acids [233].

Although numerous genes and signaling pathways have been characterized and the roles of several plant hormones have been extensively studied in stress responses in plants, the underlying molecular mechanisms are largely unknown. This is, primarily, because of the complex interactions between multiple signaling pathways [234]. Chinnusamy *et al.* [235], Xiong *et al.* [236], and Zhu [199] all reported that salinity-induced activation of many abscisic acid biosynthetic genes, such as zeaxanthin oxidase, 9-cis-epoxycarotenoid dioxygenase, abscisic acid-aldehyde oxidase, and molybdenum cofactor sulfurase, appear to be regulated through a calcium-dependent phosphorylation pathway. In the same sense, a molecular link between auxin signaling and salt stress has been established by Jung and Park [237]. These authors suggest that a membrane bound transcription factor (NTM2) incorporates auxin signal in seed germination which modulates seed germination under salinity stress.

## 6. Concluding Remarks

The recent knowledge reported in this review highlights legumes’ potential to cope with the most abundant abiotic constraints worldwide, through key mechanisms including constitutive and stress-induced responses. Advanced knowledge at the morphological, physiological and molecular level has enabled breeding for candidate genes and genetic engineering for legume crops that are better adapted to stressful conditions. In spite of this, the extent of the stress tolerance in plants largely depends on factors that vary among genotypes and environmental conditions, as well as the complexity and severity of the imposed stress. The last factor also includes the situation where multiple abiotic constraints occur at the same time, which highlights the important challenges that research in plant breeding need to address in order to develop legumes with tolerance to multiple constraints.

Coincident with most knowledge on abiotic factors and their effects on plant performance, there is an increasing awareness and interest for exploration of biotic factors with synergistic and complementary interactions for the benefit of both plants and soil microorganisms. For instance, in drought-stressed legumes, a positive relationship has been found between efficient rhizobial symbiosis and osmotic stress tolerance, indicating that efficient nodulation confers plant drought tolerance in terms of growth, improvement of plant water status and alleviation of oxidative stress [238]. Increased P-deficiency tolerance and its use efficiency may occur through naturally-formed mycorrhizal symbioses or through genetically-modified crops. The latter is exemplified by the induced-organic acids’ exudation in plants that have been transformed for efficient P uptake, which was shown to be a particularly valuable trait if accompanied by the ability to release compounds that prevent any further microbial breakdown of organic acids in the rhizosphere [68,239]. Enhanced P availability may also improve tolerance to toxic elements, as indicated by the findings that P and Cd treated roots of *Trifolium* sp. produced polyphosphate which chelated Cd in the mycorrhizal hyphae of *Rhizophagus irregularis*, with improved fitness for both the plant and fungal partners of the symbiosis [240]. Also, positive effects on osmotic adjustment under drought and salinity stress have often been associated with ectomycorrhizal colonization [241]. However, while these highly valuable mycorrhizae biotechnological applications are well known for tree improvement, significant progress is still lacking in the use of mycorrhizae for improving legumes performance under environmental constraints.

Another interesting approach is the design and use of novel diversity-based cropping systems considering legumes as a main component in order to increase both above- and below-ground functional diversity. Diversified cropping through increasing intra- and inter-specific diversity will in many cases have positive impacts on resilience to abiotic and biotic stress [242,243,244], resource use efficiency, soil fertility, and yield stability [245,246]. Therefore, more concerted and holistic efforts at the cropping system level, including breeding for stress tolerance, choosing appropriate varieties under the given environmental conditions and combining traits in mixed crops to sustain yields under stressful conditions, should be promoted. However, increasing crop diversity would make it difficult to reveal traits in complex belowground interactions that include microbial communities, root architecture and plasticity, nodule development and distribution. Therefore, advanced studies are needed to understand the mechanisms of induced rhizosphere heterogeneity in diversified crops, aiming to optimize the trait complementarity among roots, nodules and rhizosphere properties for improved stress tolerance.

By presenting the current state of the art for the most important abiotic stress factors and responses in legume and their SNF, we hope that this review will serve as a resource of knowledge for further improvements of crop production under environmental constraints. Plant production in suboptimal conditions often imposes a combination of several abiotic and biotic stress factors. In this context, we highlight two central strategies for the future advancement of knowledge and innovation, targeting the multiple challenges of legume production under environmental constraints: (1) identifying legume genotypes and symbiotic combinations that combine multiple stress tolerance traits, and applying these in practice; (2) improving the understanding of above- and below-ground interactions in diversified crops, for the development of cropping systems that optimize complementary or facilitating mechanisms, thereby improving yield stability.
